# Scaling Pharmacodynamics from Rats to Humans to Support Erythropoietin and Romiplostim Combination Therapy to Treat Erythropoietin-Resistant Anemia

**DOI:** 10.3390/pharmaceutics15020344

**Published:** 2023-01-19

**Authors:** Xiaoqing Fan, Wojciech Krzyzanski, Dongyang Liu, Raymond S. M. Wong, Xiaoyu Yan

**Affiliations:** 1 School of Pharmacy, Faculty of Medicine, The Chinese University of Hong Kong, Shatin, Hong Kong SAR, China; 2 Department of Pharmaceutical Sciences, The State University of New York at Buffalo, Buffalo, NY 14260, USA; 3 Drug Clinical Trial Center, Peking University Third Hospital, Beijing 100191, China; 4 Division of Hematology, Department of Medicine and Therapeutics, Faculty of Medicine, The Chinese University of Hong Kong, Shatin, Hong Kong SAR, China

**Keywords:** erythropoietin, romiplostim, pharmacodynamics, allometric scaling, rats, humans

## Abstract

Recombinant human erythropoietin (rHuEPO) is one of the most effective drugs for the treatment of anemia in patients with chronic kidney disease. However, EPO-resistance is an important contributor to the increased risk of adverse effects. We previously showed that EPO treatment could induce precursor cell depletion, resulting in EPO-resistance. We further found that the combination of EPO with romiplostim, a thrombopoietin receptor agonist that can stimulate the expansion of hematopoietic stem cells, can treat EPO-resistance. In this study, we performed interspecies pharmacodynamic (PD) scaling of this combination therapy for human dose prediction. The pharmacokinetic parameters of both rHuEPO and romiplostim in humans were obtained from previous studies. The PD parameters obtained in rats were scaled to humans using allometric equations. The relationship between PD parameters of the megakaryocyte lineage from rats, monkeys, and humans was in agreement with those from the literature on allometric scaling. The PD response was translated to humans based on allometric scaling and agreed with the observed data. These parameters were used to simulate hemoglobin and platelet response in humans. RHuEPO 50 IU/kg thrice weekly and romiplostim 1 μg/kg once every 4 weeks from the second week is the recommended combination dosing regimen according to the model prediction. Our work successfully scaled the PD of rHuEPO and romiplostim monotherapy from rats to humans. The predicted dosing regimen of each drug in the combination therapy is less intensive than the approved starting dose of each drug, which supports additional evaluations of the combination therapy in humans.

## 1. Introduction

Anemia is a common complication of chronic kidney disease (CKD) and is associated with increased cardiovascular events and hospitalizations. Erythropoietin (EPO) deficiency is considered a primary etiologic factor for renal anemia [1]. Recombinant human erythropoietin (rHuEPO) has revolutionized anemia management, as it reduces the need for red blood cell (RBC) transfusions and improves anemia-related symptoms and quality of life [2,3]. Despite the great efficacy of rHuEPO, randomized controlled trials have shown that high-dose erythropoiesis-stimulating agents (ESAs) increase the risks of mortality and cardiovascular events [4,5,6]. Therefore, the United States Food and Drug Administration has recommended that the lowest possible erythropoiesis-stimulating agent dose be used when treating patients on hemodialysis for end-stage renal disease. In addition, up to 10% of CKD patients with anemia fail to achieve the hemoglobin (Hgb) target after rHuEPO treatment or require large doses of rHuEPO to maintain a target Hgb concentration. These patients are recognized as EPO-resistant or hyporesponsive [7] and are typically switched back to blood transfusion, which may lead to poor compliance and associated side effects [8,9,10,11]. Therefore, it is essential to develop novel and effective approaches to stimulate erythropoiesis, reduce the need for erythropoiesis-stimulating agent doses and transfusions, and correct EPO resistance.

Our previous study showed that intensive rHuEPO treatment can induce erythroid precursor cell depletion, resulting in EPO resistance [12], and we further reported that a combination of thrombopoietin receptor agonists (TPO-RAs) with EPO can promote erythropoiesis synergistically [13,14]. Moreover, we demonstrated that EPO can drive the fate of bipotent megakaryocyte–erythroid progenitors (MEPs) toward the erythroid lineage, thus restoring the platelet count to the normal physiological range through MEP competition and reducing the risk of thrombosis. These results support the use of EPO in combination with romiplostim, a second-generation thrombopoietin receptor agonist that can stimulate the expansion of hematopoietic stem cells, in treating EPO resistance.

It is important to ascertain how data obtained from animal models can be extrapolated quantitatively to humans. Allometric scaling has been successfully used for cross-species scaling of pharmacokinetics (PK) and pharmacodynamics (PD) [15,16,17,18]. It is based on the concept that the physiological and biochemical variables of different mammals are similar across species with respect to physiological factors such as body weight (BW) when handling certain drugs [19,20].

The objectives of this study were to scale the previously developed PK/PD model of rHuEPO and romiplostim combination therapy from rats to humans. The scaled PD responses were compared with observed effects in humans for validation. The validated PK/PD model was used to predict the combination dosing regimen in humans to support future clinical trials.

## 2. Materials and Methods

### 2.1. Michaelis–Menten Approximation of Target-Mediated Drug Disposition PK/PD Model Development

**Figure 1:**
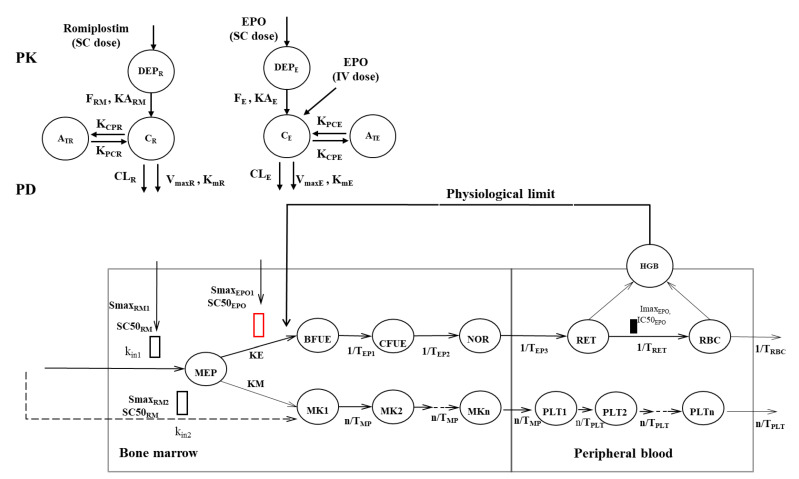
Schematic diagrams of the proposed PK/PD model of the effects of romiplostim and rHuEPO on red blood cells and platelet production. The open rectangle indicates the stimulatory effects of romiplostim (black) and rHuEPO (red). The solid rectangle indicates the inhibitory effect of rHuEPO. DEP_E_ and DEP_R_ are the absorption compartments for rHuEPO and romiplostim, respectively. A_TE_ and A_TR_ are the peripheral compartments for rHuEPO and romiplostim, respectively. C_E_ and C_R_ are the central compartments for rHuEPO and romiplostim, respectively. KA_E_ and KA_RM_ are the absorption rates of rHuEPO and romiplostim, respectively; F_E_ and F_RM_ are the bioavailabilities of rHuEPO and romiplostim, respectively; CL_EPO_ and CL_RM_ are the linear clearances of rHuEPO and romiplostim from the central compartment, respectively. V_maxE_ and K_mE_ denote the maximum elimination rate and Michaelis constant of rHuEPO, respectively; V_maxR_ and K_mR_ denote the maximum elimination rate and Michaelis constant of romiplostim, respectively, which were used to describe Michaelis–Menten saturable kinetics; and K_CPE_ and K_PCE_ are the intercompartmental rate constants of rHuEPO. K_CPR_ and K_PCR_ are the intercompartmental rate constants of romiplostim. SC = subcutaneous, IV = intravenous, T_EP_ represents the average time required for precursors to develop into the next cell population. T_RET_ and T_RBC_ represent the mean residence time for reticulocytes (RETs) and mature red blood cells (RBCs), respectively. Smax_EPO1_, Smax_RM1_, and Smax_RM2_ are the maximum stimulatory effect of rHuEPO and romiplostim, respectively. SC_50_ and IC_50_ = drug concentrations that induce half-maximum effect; Imax = maximum inhibitory effect. The series of n = 10 aging compartments (MKi, i = 1, …, n) denotes the MK precursor cells, with the first-order transition rates n/TMP; PLTi (i = 1, …, n) represents the platelets with the transition rates n/TPLT. BFUE = burst forming unit-erythroid cells, CFUE = colony-forming unit-erythroid cells, NOR = normoblasts. Kin1 and Kin2 are zero-order rate constants for producing MEP and MK1, respectively. MEPs proliferate to erythroid and MK lineages according to the first-order rate constant KE and KM, respectively.

The mechanism-based PK/PD model structure was described in our previous publication [13]. The rHuEPO and romiplostim PK parameters in humans were estimated using the target-mediated drug disposition (TMDD) model [21,22], whereas the PK model described in our previous publication was a two-compartment model for rHuEPO and one-compartment model for romiplostim [13]. To adjust the compartmental PK model to the target-mediated drug disposition model for more precise allometric scaling, Michaelis–Menten (M-M) approximation of the target-mediated drug disposition model was incorporated into our mechanism-based PK/PD model. The general structure of the Michaelis–Menten-target-mediated drug disposition PK/PD model of rHuEPO and romiplostim is shown in Figure 1. The PK model for rHuEPO and romiplostim consists of an Michaelis–Menten approximation of the target-mediated drug disposition model, assuming quasi-equilibrium. The differential equations for rHuEPO PK after intravenous (IV) or subcutaneous (SC) administration are as follows (Equations (1)–(6)):

(1) $$\frac{{\mathrm{dA}}_{\mathrm{DEPE}}}{\mathrm{dt}}=-{\mathrm{KA}}_{E}\cdot A_{\mathrm{DEPE}}{\;\mathrm{where}\;A}_{\mathrm{DEPE}}\left(0\right)=F_{E}*\mathrm{Dose}\_{\mathrm{SC}}_{E}$$

(2) $$\frac{{\mathrm{dA}}_{\mathrm{CEPO}}}{\mathrm{dt}}={\mathrm{KA}}_{E}\cdot A_{\mathrm{DEPE}}-{\mathrm{CL}}_{\mathrm{EPO}}\cdot C_{\mathrm{EPO}}-\frac{V_{\mathrm{MEPO}}\cdot C_{\mathrm{EPO}}}{K_{\mathrm{MEPO}}+C_{\mathrm{EPO}}}-K_{\mathrm{CPEPO}}\cdot V_{\mathrm{CEPO}}\cdot C_{\mathrm{EPO}}+K_{\mathrm{PCEPO}}\cdot A_{\mathrm{PEPO}}\;\mathrm{where}{\;A\;}_{\mathrm{CEPO}}\left(0\right)=\mathrm{Dose}\_{\mathrm{IV}}_{E}$$

(3) $$\frac{{\mathrm{dA}}_{\mathrm{PEPO}}}{\mathrm{dt}}=K_{\mathrm{CPEPO}}\cdot V_{\mathrm{CEPO}}\cdot C_{\mathrm{EPO}}-K_{\mathrm{PCEPO}}\cdot A_{\mathrm{PEPO}}\;{\mathrm{where}\;A}_{\mathrm{PEPO}}\left(0\right)=0$$

(4) $$C_{\mathrm{EPO}}=\frac{1}{2}\times (C_{\mathrm{TOTE}}-R_{\mathrm{TOTE}}-k_{\mathrm{MEPO}}+\sqrt{{\left(C_{\mathrm{TOT}}-R_{\mathrm{TOTE}}-k_{\mathrm{MEPO}}\right)}^{2}+4\times k_{\mathrm{MEPO}}\times C_{\mathrm{TOTE}}}$$

(5) $$R_{\mathrm{TOTE}}=V_{\mathrm{MEPO}}/\left(V_{\mathrm{CEPO}}\times k_{\mathrm{INTE}}\right)$$

(6) $$C_{\mathrm{TOTE}}=A_{\mathrm{CEPO}}/V_{\mathrm{CEPO}}$$

Similarly, the differential equations for romiplostim PK after SC administration are as follows (Equations (7)–(12)):

(7) $$\frac{{\mathrm{dA}}_{\mathrm{DEPR}}}{\mathrm{dt}}=-{\mathrm{KA}}_{\mathrm{RM}}\cdot A_{\mathrm{DEPR}}{\;\mathrm{where}\;A}_{\mathrm{DEPR}}\left(0\right)=F_{\mathrm{RM}}*\mathrm{Dose}\_{\mathrm{SC}}_{\mathrm{RM}}$$

(8) $$\frac{{\mathrm{dA}}_{\mathrm{CRM}}}{\mathrm{dt}}={\mathrm{KA}}_{\mathrm{RM}}\cdot A_{\mathrm{DEPR}}-{\mathrm{CL}}_{\mathrm{RM}}\cdot C_{\mathrm{RM}}-\frac{V_{\mathrm{MRM}}\cdot C_{\mathrm{RM}}}{K_{\mathrm{MRM}}+C_{\mathrm{RM}}}-K_{\mathrm{CPRM}}\cdot V_{\mathrm{CRM}}\cdot C_{\mathrm{RM}}+K_{\mathrm{PCRM}}\cdot A_{\mathrm{PRM}}\;\mathrm{where}{\;A}_{\mathrm{CRM}}\left(0\right)=\mathrm{Dose}\_{\mathrm{IV}}_{\mathrm{RM}}$$

(9) $$\frac{{\mathrm{dA}}_{\mathrm{PRM}}}{\mathrm{dt}}=K_{\mathrm{CPRM}}\cdot V_{\mathrm{CRM}}\cdot C_{\mathrm{RM}}-K_{\mathrm{PCRM}}\cdot A_{\mathrm{PRM}}{\mathrm{where}\;A}_{\mathrm{PRM}}\left(0\right)=0$$

(10) $$C_{\mathrm{RM}}=\frac{1}{2}\times (C_{\mathrm{TOTR}}-R_{\mathrm{TOTR}}-k_{\mathrm{MRM}}+\sqrt{{\left(C_{\mathrm{TOTR}}-R_{\mathrm{TOTR}}-k_{\mathrm{MRM}}\right)}^{2}+4\times k_{\mathrm{MRM}}\times C_{\mathrm{TOTR}}}$$

(11) $$R_{\mathrm{TOTR}}=V_{\mathrm{MRM}}/\left(V_{\mathrm{CRM}}\times k_{\mathrm{INTR}}\right)$$

(12) $$C_{\mathrm{TOTR}}=\frac{A_{\mathrm{CRM}}}{V_{\mathrm{CRM}}}$$

where A_DEPE_, A_CEPO_, and A_PEPO_ are the amounts of rHuEPO in the absorption, central, and peripheral compartments, respectively, and A_DEPR_, A_CRM_, and A_PRM_ are the amounts of romiplostim in the absorption, central, and peripheral compartments, respectively. KA_E_ and KA_RM_ are the absorption rates of rHuEPO and romiplostim, respectively; F_E_ and F_RM_ are the bioavailabilities of rHuEPO and romiplostim, respectively; V_CEPO_ and V_CRM_ are the volumes of the central compartments of rHuEPO and romiplostim, respectively; CL_EPO_ and CL_RM_ are the linear clearances of rHuEPO and romiplostim from the central compartment, respectively; and R_TOTE_ and R_TOTR_ represent the total EPO and TPO receptor concentrations, respectively. V_MEPO_ and K_MEPO_ denote the maximum elimination rate and Michaelis constant of rHuEPO, respectively; V_MRM_ and K_MRM_ denote the maximum elimination rate and Michaelis constant of romiplostim, respectively, which were used to describe Michaelis–Menten saturable kinetics; C_EPO_ and C_RM_ are the free serum concentrations of rHuEPO and romiplostim at time t, respectively; and K_CPEPO_ and K_PCEPO_ are the intercompartmental rate constants of rHuEPO. K_CPRM_ and K_PCRM_ are the intercompartmental rate constants of romiplostim; and K_INTE_ and K_INTR_ are the rate constants of the EPO–receptor complex and TPO–receptor complex internalization, respectively.

The previously developed PD model, which mimics the process of erythropoiesis and thrombopoiesis from bone marrow progenitor cells (MEPs) to peripheral blood cells (red blood cells and platelets), was applied directly [13]. The model is based on cell lifespan concepts by using the catenary indirect response model [23]. Details about the PD model equations were described in the original publication (provided in the Supplementary Materials).

### 2.2. Allometric Scaling and Validation

To translate the findings for combination therapy in rats to humans and to predict the optimal human dosing regimen, allometric scaling and model-based simulation were performed. Allometric scaling is based on the concept that many physiological processes and organ sizes (θ) tend to obey a power law [15]:

(13) $$\theta =a\cdot W^{b}$$

where W represents BW, and a and b are drug/process coefficients. Allometric scaling has been widely used to predict PK and PD parameters by performing least-squares linear regression to the power-based simple allometric equation.

As human PK models are available for both romiplostim and rHuEPO, they were used to drive PD in simulations directly. A brief description of the PK parameters of rHuEPO after IV or SC injection and romiplostim after SC injection in humans is presented in Table 1 [21,22,24]. Allometric scaling of rHuEPO from rats to humans has been investigated and was used in this study [25]. The PD data of romiplostim for various species were obtained from the literature [22,26]. The above relationships were established based on the data collected from healthy rats, monkeys, and humans. Then, the PD parameter estimates in rats [14,26] were used to calculate the PD parameters in humans according to the relationships. Because of the influence of disease status, it is risky to directly scale PD parameters from rats with CKD to human patients with CKD. The PD parameters in healthy rats from previous publications were used for scaling to predict the combination dosing regimen in healthy humans. The lifespan of each cell population was scaled using the allometric scaling rule. Physiological parameters such as the baseline platelet and red blood cell values were based on human values [27,28]. System-specific parameters, such as capacity (S_max_) and sensitivity (SC_50_) parameters, were directly adopted from rats and applied to humans because these parameters tend to be similar across species [15]. Only nominal variability was assigned to the baseline terms RBC_0_ and PLT_0_ (10% CV%) [15].

The scaled model for healthy subjects was validated externally using the human PD data for romiplostim and rHuEPO in the literature [21,22,24,29,30].

### 2.3. Model-Based Simulation of rHuEPO IV and Romiplostim SC Administration PD in Humans

To predict the optimal combination therapy dosing regimen in humans, the final model was used to simulate the PD profile of rHuEPO IV and romiplostim SC administration. Different dosing regimens of rHuEPO and romiplostim were considered based on the standard treatment of rHuEPO (50 IU/kg thrice weekly [TIW]) and romiplostim (1 μg/kg). Eight dosing regimens of rHuEPO and romiplostim combination therapy (Table 2) were proposed. The primary safety concern when using romiplostim to correct EPO resistance is the risk of thrombosis. The normal platelet range in healthy individuals is 0.15 to 0.35 × 10^12^/L [28]; therefore, the safety margin of 0.35 × 10^12^/L for platelets was proposed.

### 2.4. Software

PK/PD model analysis was performed using NONMEM 7.5 (Icon Development Solutions, Ellicott City, MD, USA). The ordinary differential equations were solved using the ADVAN13 subroutine, and the first-order conditional estimation method with interaction was used for all runs. The use of NONMEM was facilitated by Perl-speaks-NONMEM (version 4.9.6, http://psn.sourceforge.net/docs.php (accessed on 20 November 2022)). Graphical visualization and model diagnostics were performed using the R program (version 4.1.1, www.r-project.org (accessed on 20 November 2022)). Mean PD value time profiles for RHuEPO and romiplostim were extracted using WebPlotDigitizer 4.5 (https://apps.automeris.io/wpd/ (accessed on 20 November 2022)).

## 3. Results

### 3.1. Michaelis–Menten Approximation of a Target-Mediated Drug Disposition PK/PD Model Reasonably Characterizes the PK and PD Profiles of Romiplostim and rHuEPO as Monotherapy and Combination Therapy

The proposed Michaelis–Menten-target-mediated drug disposition PK model captured the concentration–time profiles of romiplostim and rHuEPO after both monotherapy and combination therapy in rats (Supplementary Figure S1, Table S1). Then, the typical PK parameters obtained from the PK modeling were used to drive the PD model.

The goodness-of-fit diagnostic plots (Supplementary Figure S2) for the final PD model suggested that the model adequately fitted the PD data in rats. The homogeneous distribution of data points around the identity line presented in the diagnostic plots indicated the absence of systematic bias. The parameter estimates of the model are presented in Supplementary Table S2. All fixed and random effect parameters were adequately estimated, with a relative standard error of less than 50%. The estimates of the hematological parameters T_RBC_ (mean residence times for mature RBCs), T_RET_ (mean residence times for reticulocytes), T_MP_ (mean lifespan of megakaryocyte cells), T_PLT_ (mean lifespan of platelets), RBC_0_ (baseline RBCs concentration), MCH (mean corpuscular hemoglobin), and PLT_0_ (baseline platelets in blood) were close to the physiological values [26,31].

### 3.2. Extrapolation and VALIDATION of the PK/PD Model to Humans

**Figure 2:**
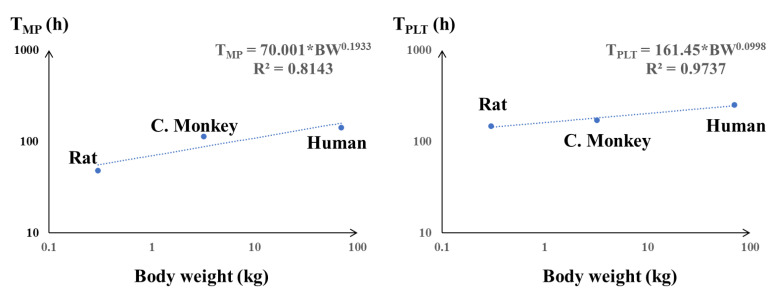
Allometric relationship of the PD parameters T_MP_ (**left panel**) and T_PLT_ (**right panel**) from rats to humans. A good correlation between body weight and the mean lifespans of megakaryocytes and platelets was observed (R^2^ > 0.8). The values of the parameters were obtained from the literature [22,26].

To further examine the model performance and translate the results from rats to humans, allometric scaling was used to extrapolate human PD parameters. The interspecies relationships of T_MP_ and T_PLT_ were described by allometric equations, as shown in Figure 2. A good correlation (R^2^ > 0.81) of body weight with T_MP_ and T_PLT_ was observed. The PD parameters from the scaling are listed in Table 3 and were retrospectively compared with the physiological values in humans. The scaled T_MP_, T_PLT_, T_RET_, and T_RBC_ were 137.1 h, 10.6 days, 44.8 h, and 119.6 days, respectively, which were close to the physiological values [22,24,32,33]. The PD parameters in humans for the scaled model are listed in Table 4.

**Figure 3:**
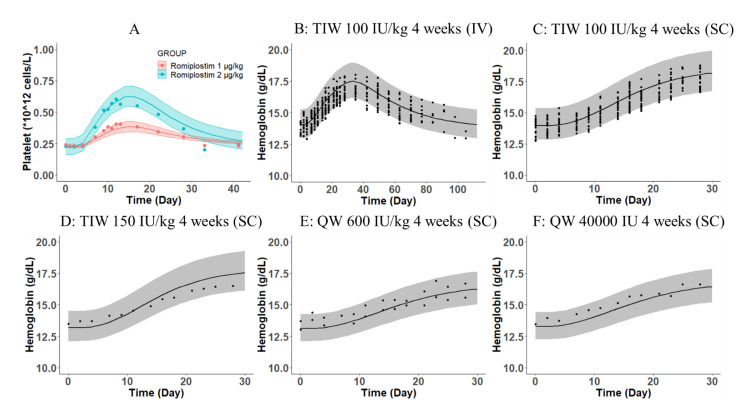
PK/PD model predicted (**A**) platelets following a single dose of romiplostim 1 or 2 μg/kg SC and hemoglobin following IV dose of rHuEPO 100 IU/kg thrice weekly (TIW) (**B**), SC dose of rHuEPO 100 IU/kg TIW (**C**), SC dose of rHuEPO 150 IU/kg TIW (**D**), SC dose of rHuEPO 600 IU/kg once weekly (QW) (**E**), and SC dose of rHuEPO 60,000 IU QW (**F**) for 4 weeks in healthy subjects. Symbols represent observed platelet and hemoglobin profiles following romiplostim or rHuEPO treatments digitized from previous reports [22,24,29,30]. The lines represent PK/PD model-predicted platelet profiles (**A**) or hemoglobin profiles (**B**–**F**) in blood. The shaded area is limited by the 20th and 80th percentiles of the 200 simulated model predictions.

Both PK parameters of the two drugs in humans were estimated using the target-mediated drug disposition model, which has been proven to adequately characterize the observed PK profiles of rHuEPO and romiplostim in humans [21,22,24]. The PD data of rHuEPO and romiplostim in healthy subjects were digitized from the original articles directly (Figure 3) [22,24,29,30]. All PK parameters of rHuEPO and romiplostim in humans were maintained identically to those derived from the previous reports (Table 1). As shown in Figure 3, the scaled PK/PD model prediction agreed well with both the observed Hgb and platelet data from rHuEPO- and romiplostim-treated healthy volunteers. In general, the translational mechanism-based PK/PD simulation adequately described the human RBC and platelet responses following repeated IV or SC administration of rHuEPO and a single SC injection of romiplostim. These results provide confidence in the predictive power of the scaled PK/PD model in humans.

### 3.3. Prediction of an Optimal Combination Dosing Regimen in Humans

**Figure 4:**
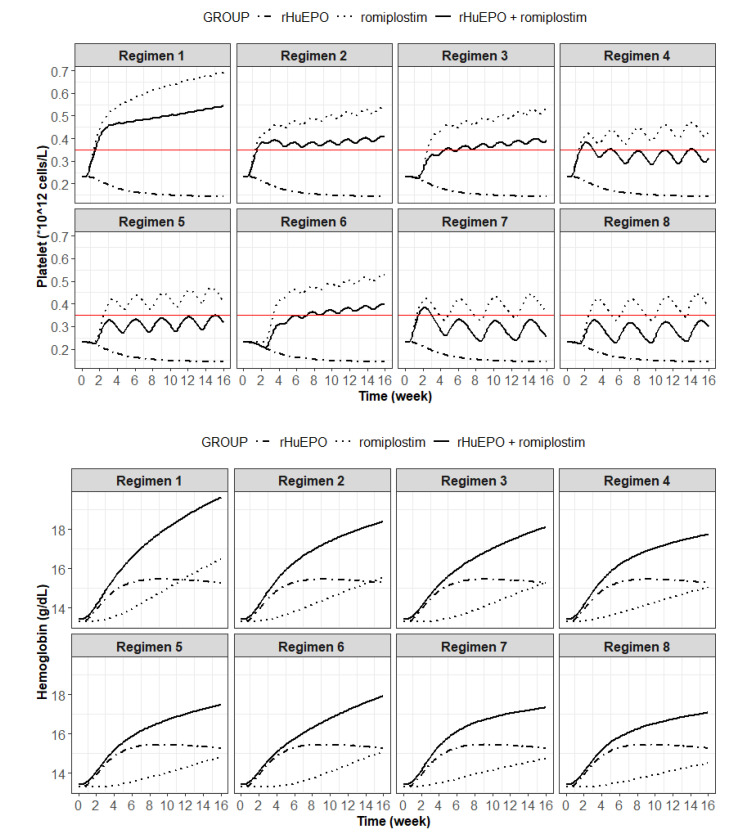
PK/PD model-predicted platelet (**upper**) and hemoglobin (**bottom**) profiles following the different dosing regimens of rHuEPO and romiplostim combination therapy in healthy subjects. The dotted, solid, and two-dash lines are the PD profiles of the rHuEPO monotherapy, rHuEPO + romiplostim combination therapy, and romiplostim monotherapy, respectively. The horizontal red line is the threshold for platelet (0.35 × 10^12^ cell/L).

**Figure 5:**
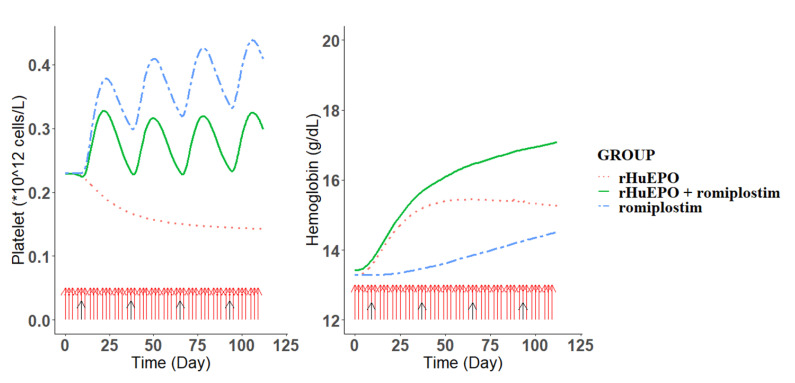
PK/PD model-predicted platelet (**left**) and hemoglobin (**right**) following the recommended dosing regimen 8 (rHuEPO 50 IU/kg thrice weekly + romiplostim 1 μg/kg once every four weeks from the second week) of rHuEPO and romiplostim combination therapy in healthy subjects. The green solid line is the PD profile of the combination therapy, whereas the red dotted line and the blue two-dash line are the corresponding rHuEPO and romiplostim monotherapy PD profiles, respectively. The arrows represent the dosing events of rHuEPO (red) and romiplostim (black).

The simulated median Hgb and platelet concentrations under eight dosing regimens of the combination therapy are shown in Figure 4, and the comparison of the simulation results is presented in Table 2. The simulation results showed that the predicted mean Hgb concentration in the rHuEPO monotherapy groups (conventional anemia treatment) reached a peak value of 15.4 g/dL on day 64 and then decreased thereafter although rHuEPO was still being administered. This result suggested EPO hyporesponsiveness, consistent with a previous report [24]. When combined with romiplostim under different dosing regimens, the Hgb concentration in all combination treatment groups was further increased, which indicated that the combination therapy could correct EPO resistance. However, the platelet count exceeded the normal limit of 0.35 × 10^12^/L in some dosing regimens (regimens 1, 2, 3, 4, 6, and 7), which was considered unacceptable due to the risk of thrombocytosis. The platelet count was maintained within the normal range in regimens 5 and 8, leading to a recommendation of regimen 8 (EPO 50 IU/kg thrice weekly + romiplostim 1 μg/kg once every 4 weeks [Q4W] from the second week) (Figure 5) for patients, given the efficacy, compliance, and cost-effectiveness.

## 4. Discussion

Our previous studies in anemic CKD rats demonstrated that romiplostim in combination with rHuEPO has great potential to correct EPO resistance [13,14]. Moreover, a mechanism-based PK/PD model was developed, which successfully quantified the interaction between rHuEPO and romiplostim. However, there is a critical gap in translating experimental data into clinical practice. Interspecies allometric scaling is a useful tool for drug development and has been frequently used to predict human PK and PD parameters [15,16,18,34,35]. To predict the optimal combination dosing regimen in humans to support future clinical trials, allometric scaling based on animal data was performed in this study.

Both rHuEPO and romiplostim are marketed drugs with clinically proven efficacy and safety. Their PK parameters in humans are available and were employed to drive the PD effects directly (Table 1). However, these parameters were estimated using the target-mediated drug disposition model, which is different from our previously developed PK model for rHuEPO and romiplostim in rats. To scale the PD parameters more accurately, the compartmental PK model was adjusted to the Michaelis–Menten target-mediated drug disposition model, and the PD parameters were re-estimated. The proposed Michaelis–Menten-target-mediated drug disposition PK/PD model (Figure 1) captured both the PK and PD profiles of romiplostim and rHuEPO after monotherapy and combination therapy, and the re-estimated PD parameters were close to the previous estimation (Table S2) [13].

Next, allometric scaling was performed and validated based on the PD parameters above and the allometric equation between rats and humans (Figure 2). Although the observed human megakaryocyte lifespan T_MP_, platelet lifespan T_PLT_, reticulocyte lifespan T_RET_, and red blood cell lifespan T_RBC_ values are available in the literature. It would be arbitrary to apply several kinds of human cell residence time to the model for translation directly. Instead, allometric scaling is to show that the fundamental assumption upon which these mechanism-based PK-PD models were built is preserved across different species. It is important to verify whether model parameters are meaningful across species [15]. Our model structure is similar to the previously reported structures that are based on human data [22,29]. So, it is necessary to prove that these parameters estimated from the same mechanism-based model structure are indeed scalable. Only by proving that this theory is correct can we use the same model structure established from animal data to predict human dosing regimen. The scaling of rHuEPO from rats to humans has been performed by others, which was applied in the present study [25]. The PD parameters of romiplostim, including the megakaryocyte lifespan T_MP_ and platelet lifespan T_PLT_ were scaled. The pharmacologic parameters, including the capacity (S_max_) and sensitivity (SC_50_) of rHuEPO and romiplostim, did not follow allometric principles; these tend to be similar across species because of the receptor density and/or structural homology between species [15,25]. The scaled PD parameters were close to the physiological values in humans (Table 3), which proved that the established PK/PD model was valuable for cross-species extrapolation. The scaled models were externally validated using rHuEPO and romiplostim PD data from healthy subjects, and the results demonstrated the accuracy of the scaled PK/PD model in humans (Figure 3). According to the general allometric scaling concept, the exponent b in the allometric equation tends to be approximately 0.75 for clearance processes, 1.0 for organ sizes or physiological volumes, and 0.25 for physiological times or the duration of physiological events [15,36]. Therefore, the exponent of cell lifespan tends to be 0.25 according to this theory. However, this type of relationship works best for drugs eliminated by direct physical processes, such as renal excretion [37]. The experience with allometric scaling of macromolecule drugs is more limited compared with small molecules. Its application to drugs exhibiting nonlinear pharmacokinetics, such as target-mediated drug disposition (TMDD) systems may be different [38]. In our study, the scaling exponents are 0.1933 for T_MP_ and 0.0988 for T_PLT_, which is close to but less than 0.25. This is consistent with the scaling results for erythropoietin on erythropoiesis, the exponents are 0.148 for T_RBC_ and 0.081 for T_RET_, respectively. The exponents were less than 0.25, but their slopes were very similar to each other [25]. Although the underlying mechanism for this difference is unclear, it is generally considered that peptide and protein drugs also exhibit allometric relationships, owing to the relative species conservation of mechanisms that control the biodistribution and elimination of such compounds [15].

Model-based simulations were conducted to optimize the combination dosing regimen and thus guide future clinical trials. The results shown in Figure 4 indicate that intensive rHuEPO treatment alone could result in EPO resistance, consistent with previous studies [24]. The combination of rHuEPO with romiplostim led to a synergistic increase in the Hgb value. However, intensive administration of romiplostim resulted in a platelet count exceeding the normal range (0.35 × 10^12^/L), which increased the risk of thrombosis. The results of simulation with regimens 5 and 8 showed that the administration of romiplostim 1 μg/kg once every three weeks or 1 μg/kg once every 4 weeks from the second week was effective in correcting EPO resistance and maintaining the platelet count within a normal range simultaneously. Based on a balance between efficacy, compliance, and cost-effectiveness for patients, regimen 8 (rHuEPO 50 IU/kg thrice weekly + romiplostim 1 μg/kg once every 4 weeks from the second week) (Figure 5) is recommended as the starting dose. Moreover, as the current dosing regimen of romiplostim in immune thrombocytopenia patients is 1–10 μg/kg once weekly, this recommendation provides a huge safety margin for dose escalation during combination treatment to boost efficacy [39].

However, it should be noted that there are some deviances in the graphics conditional weighted residual (CWRES) vs. time in the initial time (Supplementary Figure S2). This may be due to the impact of the early release of reticulocytes into circulation with the stimulation of EPO [40]. Our model only described the major mechanisms controlling the production of reticulocytes and the pharmacological effects of rHuEPO on these processes. The inclusion of other processes would require additional data/parameters, which would increase the number of model parameters and subsequently impact their identifiability and precisions. So, only the major erythropoietic processes were considered in the present PK-PD model to avoid model overparameterization. Despite the limitation, the effect of EPO on the release of RETs is independent of its effect on erythroid precursor cells. These RETs tend to be destroyed and removed by the spleen through neocytolysis due to immaturity in cell membrane structure, which was considered to have less impact on the final hemoglobin production [41,42]. While our study mainly focused on the prediction of Hgb and platelet response, major mechanisms for the production of Hgb and platelet were included in this model. The predictions for Hgb and platelet were validated using the clinical PD data. We believe the model can be helpful for the dose selection of combination therapy.

There are no PK comparisons of romiplostim/rHuEPO between the disease population and healthy volunteers available in the literature at present to our knowledge. For PD parameters, the lifespan of red blood cells in the disease population (T_RBC_ = 60–112 days) would be shorter than healthy volunteers (T_RBC_ = 120 days) [43]. The shorter lifespan would not influence the results obtained here because the mechanisms of drugs’ action are independent of the cell lifespan. Currently, studies evaluating the allometric scaling of integrated PK/PD characteristics of protein drugs are relatively limited compared with small molecules. Our study may be useful for cases in which several macro-molecule drugs act on the same pharmacological process, as certain system-specific properties in the model can be shared between drugs [37].

Interestingly, there are two case reports on the combined usage of romiplostim and darbepoetin, a second-generation ESA with a longer half-life. In one case report, a patient with myelodysplastic syndrome was treated concomitantly with darbepoetin (500 μg once every three weeks for 3 months, followed by 500 μg once every two weeks for another 11 months, 300 μg once every three weeks for another 6 months, and 300 μg once every 4 weeks for another 4 months before stopping) and romiplostim (10 μg/kg once weekly for 9 months) [44]. The results of that case were consistent with our preclinical results, and romiplostim was suggested to stimulate the erythroid response in addition to the effects of darbepoetin and a reduced darbepoetin dosage. Meanwhile, the platelet count did not increase during the combined use of darbepoetin and romiplostim [44]. Because TPO-RAs have shown efficacy in this patient population [45], the observation by Prica and Buckstein supports the inhibitory role of darbepoetin on platelets in combination therapy. The other case report showed that the combination of romiplostim and darbepoetin was successfully used as supportive therapy for chemotherapy-associated anemia and thrombocytopenia during induction chemotherapy in a patient with acute lymphoblastic leukemia who wished to avoid blood transfusions due to their beliefs as a Jehovah’s Witness [46].

It should be noted that the actual dosing regimen needs to be adjusted according to the clinical situation. According to the drug label of epoetin, the recommended starting dose for adult patients with CKD is 50–100 U/kg thrice weekly IV or SC, and the IV route is recommended for patients on hemodialysis [47]. The model validation results (Figure 3) supported the similar efficacy of the SC dosing regimen, which could also be considered as a combination therapy in a future clinical trial. Moreover, the dose of rHuEPO should be adjusted (reduced by 25%) if the Hgb concentration rises rapidly (e.g., >1 g/dL over 2 weeks) to reduce rapid responses. When combined with romiplostim, increases in Hgb should be monitored, and the dose of rHuEPO should be adjusted if Hgb increases too rapidly (>1 g/dL over 2 weeks). Given the dose titration algorithm, the doses of TPO-RAs and ESAs might shift in the same direction during titration to inhibit platelet production, according to the mechanisms of action of the combination therapy.

This study has one limitation. Given the limited dataset included in the analysis, the lack of access to individual data, and the intersubject variability in platelets and RBCs, whether the relationship between response and combination therapy holds for other dosing regimens under different scenarios remains to be further examined. Nevertheless, the mechanistic nature of this target-mediated drug disposition-PK/PD model renders it a valuable tool for developing optimal dosing regimens in the clinic.

In summary, the use of interspecies allometric scaling, values of clinical drug-specific and physiological system-specific PK and PD parameters from the literature, and a PD simulation allowed for extrapolation of experimental data to humans with a reasonable degree of success. The established PK/PD model was able to be utilized to predict the PD responses of romiplostim and rHuEPO in both monotherapy and combination therapy in healthy subjects. These data enable a recommendation for an optimal a combination dosing regimen to treat EPO resistance and help to guide subsequent studies as a starting dose in a dosing-finding study for patients with erythropoietin-resistant anemia. Furthermore, different formulations and generations of ESAs, including the short-acting epoetin and long-acting darbepoetin, and TPO-RAs, such as eltrombopag and avatrombopag, are available. The established PK/PD model could help to facilitate the clinical development of different strategies involving combinations of other ESAs and TPO-Ras and can be used to select starting doses in the treatment of CKD anemia. Further evaluation is warranted.

## Figures and Tables

**Table 1:** PK parameters of rHuEPO and romiplostim in humans obtained from the literature [21,22,24].

Parameter (Unit)	Description	Value	References
CL_E_ (L/h)	Clearance of rHuEPO	0.379	[21,24]
KA_E_ (1/h)	Absorption rate of rHuEPO	0.0269	
F_E_	Bioavailability of rHuEPO	0.513	
V_2E_ (L)	Volume of distribution of the central compartment of rHuEPO	3.25	
V_3E_ (L)	Volume of distribution of the peripheral compartment of rHuEPO	1.64	
Q_E_ (L/h)	Tissue distribution clearance of rHuEPO	0.0993	
RTOT (IU/L)	Baseline total receptor	154.7	
KM_E_ (IU/L)	Michaelis constant of rHuEPO	48.1	
KINT_E_ (1/h)	Internalization rate constant of rHuEPO	0.171	
KDEG_E_ (1/h)	Degradation rate constant	0.392	
CL_R_ (L/h)	Clearance of romiplostim	0.183	[22]
V_2R_ (L)	Volume of distribution of the central compartment of romiplostim	4.781	
K_CPR_ (1/h)	Intercompartment rate constant of romiplostim	0.0806	
K_PCR_ (1/h)	Intercompartment rate constant of romiplostim	0.0148	
KA_RM_ (1/h)	Absorption rate of romiplostim	0.0254	
F_RM_	Bioavailability of romiplostim	0.499	
KM_R_ (ng/mL)	Michaelis constant of romiplostim	0.131	
ξ_R_ (fg/platelet)	Total c-Mpl receptor concentration	0.0215	
KINT_R_ (1/h)	Internalization rate constant of romiplostim	0.173	

**Table 2:** Model-based prediction summary in the combination therapy group. The dosing regimen for rHuEPO is 50 IU/kg thrice weekly IV for 16 weeks (102 days) and that of romiplostim is 1 μg/kg SC according to the package insert. The criterion for the prediction results is a platelet range within 0.15–0.35 × 10^12^/L compared with healthy individuals. QW = once weekly, Q2W = once every two weeks, Q3W = once every three weeks, Q4W = once every four weeks.

Regimen Number	Dosing Regimen	Results	Comments
1	Romiplostim QW 1 μg/kg for 16 weeks	Platelet count exceeds 0.35 × 10^12^/L on day 11	Unacceptable
2	Romiplostim 1 μg/kg Q2W from the first week (weeks 1, 3, 5, 7, 9, 11, 13, 15)	Platelet count exceeds 0.35 × 10^12^/L on day 11	Unacceptable
3	Romiplostim 1μg/kg Q2W from the second week (weeks 2, 4, 6, 8, 10, 12, 14, 16)	Platelet count exceeds 0.35 × 10^12^/L on day 31	Unacceptable
4	Romiplostim 1 μg/kg Q3W from the first week (weeks 1, 4, 7, 10, 13, 16)	Platelet count exceeds 0.35 × 10^12^/L on day 11	Unacceptable
5	Romiplostim 1 μg/kg Q3W from the second week (weeks 2, 5, 8, 11, 14)	Platelet count will not exceed 0.35 × 10^12^/L	Acceptable
6	Romiplostim 1 μg/kg Q2W from the third week (weeks 3, 5, 7, 9, 11, 13, 15)	Platelet count exceeds 0.35 × 10^12^/L on day 50	Unacceptable
7	Romiplostim 1 μg/kg Q4W from the first week (weeks 1, 5, 9, 13)	Platelet count exceeds 0.35 × 10^12^/L on day 11	Unacceptable
8	Romiplostim 1 μg/kg Q4W from the second week (weeks 2, 6, 10, 14)	Platelet count will not exceed 0.35 × 10^12^/L	Acceptable (Recommended)

**Table 3:** Estimated PD parameters in healthy rats, scaled PD parameters in healthy subjects using the allometric equation, and observed PD parameters in healthy subjects from the literature [14,22,24,26,27,28,29,32,33].

Parameter	Unit	Estimated Value (Rat)	Scaled Value (Humans)	The Observed Value in Humans
T_MP_	h	47.8 [26]	137.1	142 [22]
T_PLT_	day	6.17 [26]	10.6	8–12 [22,33]
PLT_0_	×10^12^ cells/L	0.869 [26]	Fixed to human value	0.23 [22,28]
T_RET_	h	20 [14]	44.8	57.3 [24,32]
T_RBC_	day	60.8 [14]	119.6	120 [29]
RBC_0_	×10^12^ cells/L	7.38 [14]	Fixed to human value	4.4 [24,27]

**Table 4:** PD parameters in humans for the scaled model.

Parameter	Parameter Explanation	Unit	Value
T_MP_	Mean lifespan of megakaryocyte cells	h	137.1 (scaled)
T_PLT_	Mean lifespan of platelets	h	254.4 (scaled)
PLT_0_	Baseline platelets in blood	×10^12^ cells/L	0.23 (fixed to human value)
T_RBC_	Mean residence time for mature RBCs	day	119.6 (scaled)
T_RET_	Mean residence time for RETs	h	44.8 (scaled)
RBC_0_	Baseline RBCs concentration	×10^12^ cells/L	4.4 (fixed to human value)
KE	First-order rate constant of MEPs differentiate into BFU-E	×10^−4^/h	6.84 (not scaled)
KM	First-order rate constant of MEPs differentiate into MK1	×10^−4^/h	1.18 (not scaled)
Smax_RM1_	Maximal stimulus of romiplostim on MEPs	Dimensionless	1.67 (not scaled)
Smax_RM2_	Maximal stimulus of romiplostim on MK-committed pathway	Dimensionless	27.8 (not scaled)
Smax_EPO1_	Maximal stimulus of rHuEPO on MEPs	Dimensionless	11.3 (not scaled)
SC50_RM_	The concentrations of romiplostim that induce a half-maximum effect	ng/mL	11.9 (not scaled)
SC50_EPO_	The concentrations of rHuEPO that induce a half-maximum effect	mIU/mL	46.9 (not scaled)
Imax_EPO_	Maximal inhibition of rHuEPO on RETs aging rates	Dimensionless	0.422 (not scaled)
IC50_EPO_	The concentration of rHuEPO that induces half-maximum inhibition	mIU/mL	5.59 (not scaled)
MCH	Mean corpuscular hemoglobin	pg/cell	30.2 (fixed to human value)

## Data Availability

The data that support the findings of this study are available from the corresponding author upon reasonable request.

## Supplementary Materials

The following supporting information can be downloaded at: https://www.mdpi.com/article/10.3390/pharmaceutics15020344/s1, Figure S1: General goodness-of-fit of the final model for rHuEPO (A) and romiplostim (B). The top panels of (A) and (B) present the observed data vs. the population predictions (left) and individual predictions (right), respectively. The bottom panels of (A) and (B) present the conditional weighted residual (CWRES) vs. the time (left) and population predictions (right), respectively. The blue lines are the loess smooth lines. The gray diagonal (top panels) and horizontal (bottom panels) lines are the identity and zero lines, respectively. Figure S2. General goodness-of-fit of the final PD model, including platelets (PLT, top panels), reticulocytes (RETs, upper middle panels), RBC counts (lower middle panels), and Hgb concentration (bottom panels). Following the left-to-right order, the panels present the observed data vs. population predictions, observed data vs. individual predictions, conditional weighted residual (CWRES) vs. time, and CWRES vs. population predictions, respectively. The blue lines are the loess smooth lines. The gray diagonal and horizontal lines are the identity and zero lines, respectively. Table S1. Model estimates of the fixed- and random-effect PK parameters together with their relative standard errors. IIV = interindividual variability. Table S2. Model estimates of the fixed- and random-effect PD parameters together with their relative standard errors (RSEs).
