# Supplementary material for: Scaling Pharmacodynamics from Rats to Humans to Support Erythropoietin and Romiplostim Combination Therapy to Treat Erythropoietin-Resistant Anemia
Source: Pharmaceutics. 2023 Jan 19;15(2):344. doi: 10.3390/pharmaceutics15020344 (PMC9962863; doi:10.3390/pharmaceutics15020344)
Supplement: Supplementary file 1 [file pharmaceutics-15-00344-s001.zip › pharmaceutics-2079045-supplementary.pdf]

Scaling pharmacodynamics from rats to humans to support erythropoietin and romiplostim combination therapy to treat erythropoietin-resistant anemia

Xiaoqing Fan[1], Wojciech Krzyzanski[2], Dongyang Liu[3], Raymond S. M. Wong[4], Xiaoyu Yan[1]*

[1]School of Pharmacy, Faculty of Medicine, The Chinese University of Hong Kong, Shatin, Hong Kong, China SAR

[2]Department of Pharmaceutical Sciences, The State University of New York at Buffalo, Buffalo, NY, USA

[3]Drug Clinical Trial Center, Peking University Third Hospital, Beijing, China

[4]Division of Hematology, Department of Medicine and Therapeutics, Faculty of Medicine, The Chinese University of Hong Kong, Shatin, Hong Kong, China SAR

*** Corresponding author:** Xiaoyu Yan, PhD

Address: 8th Floor, Lo Kwee-Seong Integrated Biomedical Sciences Building, Area 39, The Chinese University of Hong Kong, Shatin, N.T. Hong Kong

Tel: +852-34935012 **Fax:** +852-26035295

Email address: xiaoyuyan@cuhk.edu.hk

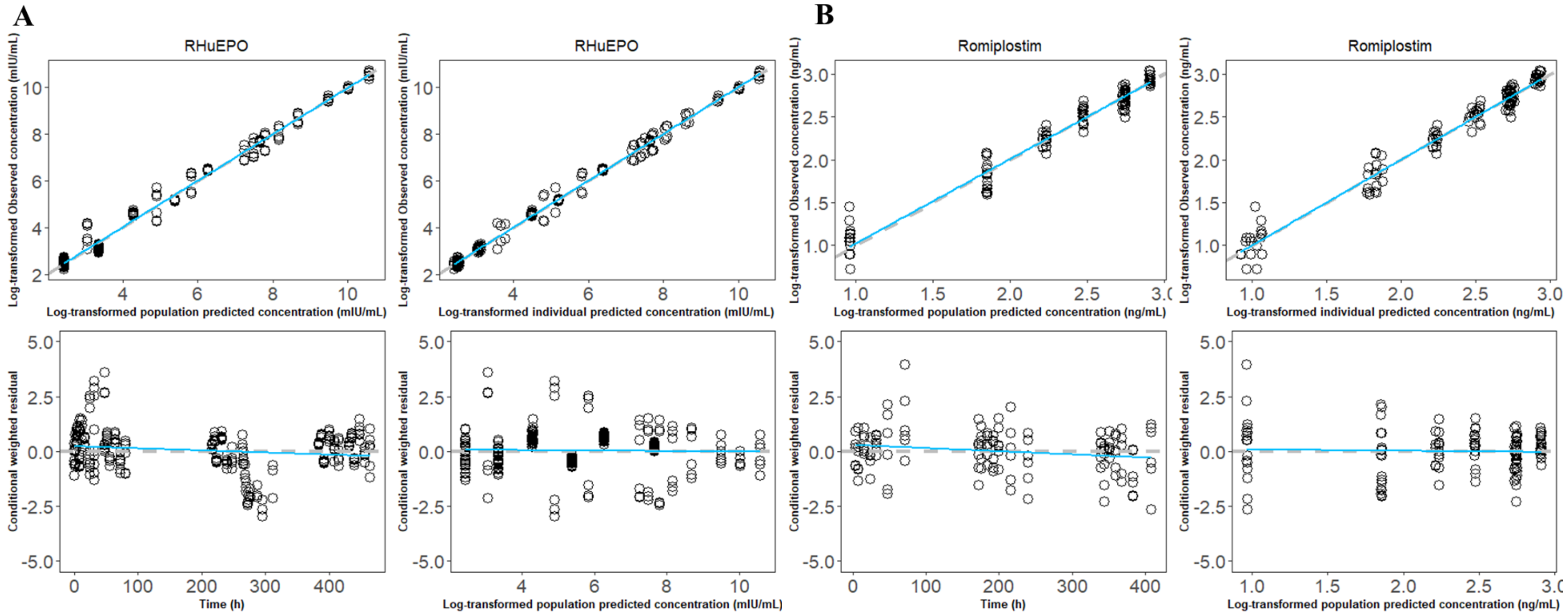

Figure S1. General goodness-of-fit of the final model for rHuEPO (A) and romiplostim (B). The top panels of (A) and (B) present the observed data vs. the population predictions (left) and individual predictions (right), respectively. The bottom panels of (A) and (B) present the conditional weighted residual (CWRES) vs. the time (left) and population predictions (right), respectively. The blue lines are the loess smooth lines. The gray diagonal (top panels) and horizontal (bottom panels) lines are the identity and zero lines, respectively.

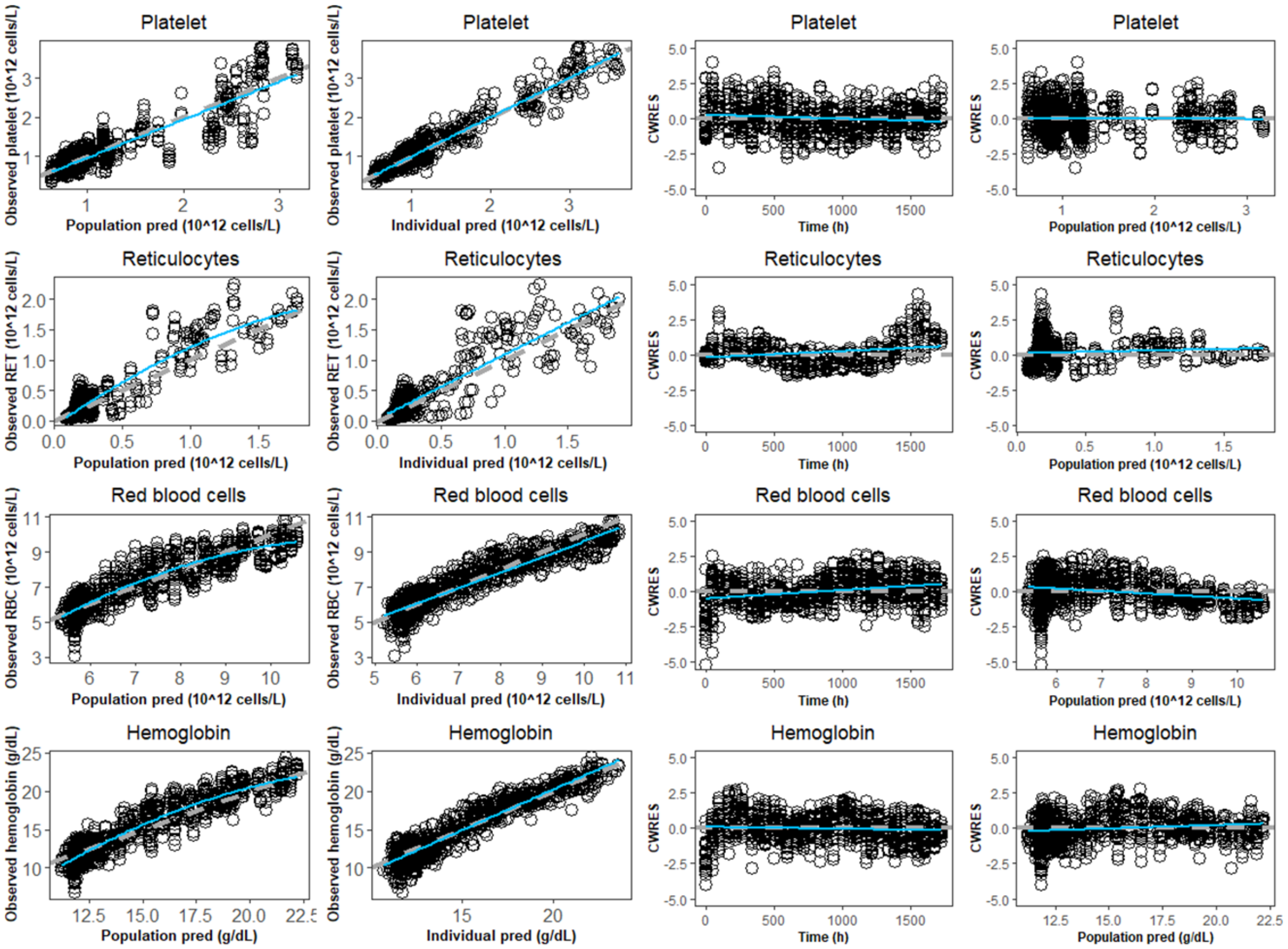

Figure S2. General goodness-of-fit of the final PD model, including platelets (PLT, top panels), reticulocytes (RETs, upper middle panels), RBC

counts (lower middle panels), and Hgb concentration (bottom panels). Following the left-to-right order, the panels present the observed data vs. population predictions, observed data vs. individual predictions, conditional weighted residual (CWRES) vs. time, and CWRES vs. population predictions, respectively. The blue lines are the loess smooth lines. The gray diagonal and horizontal lines are the identity and zero lines, respectively.

Table S1. Model estimates of the fixed- and random-effect PK parameters together with their relative standard errors. IIV = interindividual variability.

Parameter	Description	Unit	Estimate	%RSE
CL_R/F	Clearance of romiplostim	L/h/kg	0.0277	5.41
V_{2R}/F	Volume of distribution of the central compartment of romiplostim	L/kg	0.515	22.3
K_{CPR}/F	Intercompartment rate constant of romiplostim	1/h	0.0136	79.4
K_{PCR}/F	Intercompartment rate constant of romiplostim	1/h	0.0493	16.5
Ka/F	Absorption rate of romiplostim	1/h	0.0917	17.5
K_{mR}/F	Michaelis constant of romiplostim	μg/L	10.62	6.06
V_{maxR}/F	Maximum elimination rate of romiplostim	μg/h/kg	0.218	13.2
K_{INTR}/F	Internalization rate constant of romiplostim	1/h	0.0279	23.4
ω_{CL}	IIV of CL_R/F	Dimensionless	0.0553	26.5
σ of romiplostim	Additive error in logarithmic domain	Dimensionless	0.112	9.49
CL_E	Clearance of rHuEPO	L/h/kg	0.0135	3.06
V_{2E}	Volume of distribution of the central compartment of rHuEPO	L/kg	0.0293	1.64
K_{CPE}	Intercompartment rate constant of rHuEPO	1/h	0.180	4.31
K_{PCE}	Intercompartment rate constant of rHuEPO	1/h	0.196	2.90
K_{mE}	Michaelis constant of rHuEPO	IU/L	7.932	2.09
V_{maxE}	Maximum elimination rate of rHuEPO	IU/h/kg	0.289	2.56
K_{INTE}	Internalization rate constant of rHuEPO	1/h	0.00173	1.02
ω_{CLE}	IIV of CL_E	Dimensionless	0.0885	79.2
ω_{KPCE}	IIV of K_{PCE}	Dimensionless	0.161	41.2
σ of rHuEPO	Additive error in logarithmic domain	Dimensionless	0.195	3.33

Note: Relative standard errors (RSE) for ω and σ are reported on the approximate standard deviation scale (standard error/variance estimate)/2. IIV is expressed as the coefficient of variation (%). σ represents variance in the residual error.

Table S2. Model estimates of the fixed- and random-effect PD parameters together with their relative standard errors (RSEs).

Parameter	Parameter explanation	Unit	Estimate (%RSE)	IIV (%RSE)
T_{MP}	Mean lifespan of megakaryocyte cells	h	37.6 (5.24)	-[a]
T_{PLT}	Mean lifespan of platelets	h	209 (3.57)	-[a]
PLT_0	Baseline platelets in blood	$\times10^{12}$ cells/L	1.17 (1.30)	0.0567 (19.9)
T_{RBC}	Mean residence time for mature RBCs	h	998 (3.29)	-[a]
T_{RET}	Mean residence time for RETs	h	50.2 (3.61)	-[a]
RBC_0	Baseline RBCs concentration	$\times10^{12}$ cells/L	5.65 (0.689)	0.03 (34.9)
KE	First-order rate constant of MEPs differentiate into BFU-E	$\times10^{-4}$/h	6.84 (4.30)	-[a]
KM	First-order rate constant of MEPs differentiate into MK1	$\times10^{-4}$/h	1.18 (4.91)	-[a]
$Smax_{RM1}$	Maximal stimulus of romiplostim on MEPs	Dimensionless	1.67 (6.77)	-[a]
$Smax_{RM2}$	Maximal stimulus of romiplostim on MK-committed pathway	Dimensionless	27.8 (6.58)	-[a]
$Smax_{EPO1}$	Maximal stimulus of rHuEPO on MEPs	Dimensionless	11.3 (7.01)	-[a]
$SC50_{RM}$	The concentrations of romiplostim that induce a half-maximum effect	ng/mL	11.9 (7.60)	-[a]
$SC50_{EPO}$	The concentrations of rHuEPO that induce a half-maximum effect	mIU/mL	46.9 (12.7)	-[a]
$Imax_{EPO}$	Maximal inhibition of rHuEPO on RETs aging rates	Dimensionless	0.422 (5.97)	-[a]
$IC50_{EPO}$	The concentration of rHuEPO that induces half-maximum inhibition	mIU/mL	5.59 (9.54)	-[a]
MCH	Mean corpuscular hemoglobin	pg/cell	21.0 (2.77)	-[a]
GAM_1	Hill factor on physiological limit	Dimensionless	1.2 (5.33)	-[a]
GAM_2	Hill factor on $SC50_{RM}$	Dimensionless	94.2 (6.25)	-[a]
σ_{PLT}	Proportional error of platelets	Dimensionless	0.138 (2.58)	-[b]
σ_{RBC}	Proportional error of RBC	Dimensionless	0.0843 (2.63)	-[b]
σ_{HGB}	Additive error of HGB	Dimensionless	1.23 (2.44)	-[b]
σ_{RET2}	Proportional error of RET	Dimensionless	0.575 (4.32)	-[b]

OBJ	Objective function value	Dimensionless	-1845	-[b]

Note: The PK parameters are fixed at their estimated values. The RSEs for ω and σ are reported on the approximate standard deviation scale (standard error/variance estimate)/2. Interindividual variability (IIV) is expressed as the coefficient of variation (%). σ represents the variance in the residual error. -[a], did not apply due to no improvement in the goodness of fit. -[b], not applicable.

PD model equations:

For RBC production, the PD model comprises a series of compartments, including MEPs, BFU-E, CFU-E, normoblasts (NORs), and RETs that eventually develop into RBCs, to mimic erythropoiesis. The stimulatory effect of romiplostim targets the production rate of MEPs, and the differentiation of MEPs into BFU-E cells is controlled by processes with the first-order rate constant KE, which can be stimulated by rHuEPO, as follows (Eq. 14):

$$\frac{dMEP}{dt} = Kin1 \cdot \left(1 + \frac{Smax_{RM1} \cdot C_{ROM}}{SC50_{RM} + C_{ROM}}\right) - KE \cdot \left(1 + \frac{Smax_{EPO} \cdot C_{EPO}}{SC50_{EPO} + C_{EPO}}\right) \cdot MEP \cdot (1 - \frac{\Delta HGB}{RH})^{GAM} - KM \cdot MEP \qquad (14),$$

where Kin1 is a zero-order rate constant for producing MEPs. C_{ROM} and C_{EPO} are the serum concentrations of romiplostim and rHuEPO at time t, respectively; S_{ROM1} and $Smax_{EPO}$ are the maximal stimuli of romiplostim and rHuEPO, respectively; and $SC50_{RM}$ and $SC50_{EPO}$ are the concentrations of romiplostim and rHuEPO that induce a half-maximum effect, respectively. MEPs differentiate into erythroid and MK lineages according to the first-order rate constants KE and KM, respectively. $(1 - \frac{\Delta HGB}{RH})^{GAM}$ represent the physiological limit, a homeostatic mechanism to maintain normal body function. ΔHGB = HGB − HGB0, where HGB0 represents the baseline HGB concentration. GAM is a power coefficient. RH is the physiological limit of HGB. The highest RH for HGB was fixed at 24 based on a previous multiple-dose rHuEPO (1350 IU/kg) PK/PD study in rats. The overall production rate of HGB then became zero, preventing the response from increasing further.

$$\frac{dBFUE}{dt} = KE \cdot \left(1 + \frac{Smax_{EPO} \cdot C_{EPO}}{SC50_{EPO} + C_{EPO}}\right) \cdot MEP \cdot (1 - \frac{\Delta HGB}{RH})^{GAM} - \frac{1}{T_{EP1}} \cdot BFUE \quad (15)$$

$$\frac{dCFUE}{dt} = 2^{MCFU} \cdot \frac{1}{T_{EP1}} \cdot BFUE - \frac{1}{T_{EP2}} \cdot CFUE \quad (16)$$

$$\frac{dNOR}{dt} = 2^{MNOR} \cdot \frac{1}{T_{EP2}} \cdot CFUE - \frac{1}{T_{EP3}} \cdot NOR \quad (17)$$

$$\frac{dRET}{dt} = \frac{1}{T_{EP3}} \cdot NOR - \frac{1}{T_{RET}} \cdot RET \cdot (1 - \frac{Imax_{EPO} \cdot C_{EPO}}{IC50_{EPO} + C_{EPO}}) \quad (18)$$

$$\frac{dMRBC}{dt} = \frac{1}{T_{RET}} \cdot RET \cdot (1 - \frac{Imax_{EPO} \cdot C_{EPO}}{IC50_{EPO} + C_{EPO}}) - \frac{1}{T_{RBC}} \cdot MRBC \quad (19),$$

where 2^{MCFU} and 2^{MNOR} are factors reflecting the number of CFU-E cells that can be produced by one BFU-E and the number of NORs that can be produced by one CFU-E cell, respectively. T_{EP} represents the average time required for precursors to develop into the next cell population. T_{RET} and T_{RBC} represent the mean residence times for RETs and mature RBCs, respectively. T_{EP} was assumed to be equal to T_{RET} to reduce the number of model parameters. rHuEPO can stimulate the early release of immature RETs from BM into peripheral blood; thus, a part of rHuEPO's effect on the distribution of RET maturation times must be attributed to the release of stress RETs. Hence, in our model, the effect of rHuEPO on the age distribution of RETs was written as $(1 - \frac{Imax_{EPO} \cdot C_{EPO}}{IC50_{EPO} + C_{EPO}})$, which is consistent with the mechanism of action and greatly improves the model fit. $Imax_{EPO}$ is the maximal inhibition of rHuEPO on RETs aging rates, and $IC50_{EPO}$ is the serum concentration of rHuEPO that induces half-maximum inhibition.

HGB concentrations were derived from the mass of RBCs, which consists of mature RBCs (MRBC) and RETs:

$$RBC = MRBC + RET \tag{20}$$

$$HGB = MCH \cdot RBC/10 \tag{21}$$

where MCH is the mean corpuscular HGB, which was estimated directly from the data. The denominator 10 converts the MCH unit to pg/cell.

For platelet production, MK1 was assumed to be generated at K_{in2} in addition to the MEP differentiation pathway; the effect of romiplostim is incorporated as a stimulus on the production of both MEPs and MK1. The MK-committed progenitor pathway stimulated by romiplostim was included in the Model:

$$\frac{dMK1}{dt} = Kin2 \cdot (1 + \frac{Smax_{RM2} \cdot C_{ROM}}{SC50_{RM} + C_{ROM}}) + KM \cdot MEP - \frac{n}{T_{MP}} \cdot MK1 \tag{22}$$

S_{ROM2} is the maximal stimulus of romiplostim on Kin2. A series of aging compartments (MKn, n = 10) denoted the MK precursor cells in BM, with the first-order transition rates n/TMP. The model equations are as follows:

$$\frac{dMK_i}{dt} = \frac{n}{T_{MP}} \cdot (MK_{i-1} - MK_i) \text{ i = 2, …, n} \tag{23}$$

$$\frac{dPLT_1}{dt} = CF \cdot \frac{n}{T_{MP}} \cdot MK_n - \frac{n}{T_{PLT}} \cdot PLT_1 \tag{24}$$

Similarly, PLTn (n = 10) represents the platelets in blood with the transition rate nPLT/TPLT:

$$\frac{dPLT_i}{dt} = \frac{n}{T_{PLT}} \cdot (PLT_{i-1} - PLT_i) \text{ i = 2, …, n} \tag{25}$$,

where T_{MP} and T_{PLP} denote the mean lifespans of precursor cells and platelets, respectively. CF represents the conversion factor equal to the average number of platelets produced by an MK and was fixed at 4000. The platelets were modeled as the sum of platelet counts in each PLT compartment:

$$PLT = PLT_1 + \ldots + PLT_n \tag{26}$$

The secondary parameters and baseline equations defined by the steady-state value can be used to reduce the number of model parameters as follows:

$$RET_0 = MRBC \cdot T_{RET}/T_{RBC} \tag{27}$$

$$NOR_0 = RET_0 \cdot T_{EP3}/T_{RET} \tag{28}$$

$$CFUE_0 = RET_0 \cdot T_{EP2}/(T_{RET} \cdot 2^{MNOR}) \tag{29}$$

$$BFUE_0 = RET_0 \cdot T_{EP2}/(T_{RET} \cdot 2^{MNOR} \cdot 2^{MCFU}) \tag{30}$$

$$MEP_0 = BFUE_0/(T_{EP1} \cdot KE) \tag{31}$$

$$MKn = T_{MP} \cdot PLT_0/(CF \cdot T_{PLT} \cdot 10) \tag{32}$$

$$K_{in1} = MEP_0 \cdot (KM + KE) \tag{33}$$

$$K_{in2} = \frac{PLT_0}{CF \cdot T_{PLT}} - MEP_0 \cdot KM \tag{34}$$

The interindividual variabilities (IIVs) of fixed-effect parameters were described by the exponential error model:

$$P_i = \theta_i \cdot \exp(\eta_{P_i}) \quad (35),$$

where P_i is the ith parameter for the individual, θ_i is the typical value (estimated population geometric mean) for P_i, and η_{pi} is an independent random variable that is normally distributed with zero mean and variance (ω^2). Random effects were added on PLT_0 and RBC_0, while for other values of θ_i, variances of random effect parameters were fixed to a small value (0.0225, i.e., 15% coefficient of variation [cv]) to improve the expectation–maximization (EM) algorithm efficiency in NONMEM.

The residual variabilities in RBC, PLT, RET, and HGB were added separately. Different residual error models were explored, including an additive error model, a proportional error model, and a combined error (proportional plus additive) model. Eventually, a combined model of residual error was applied and described as:

$$Y_{ij} = \hat{Y}_{ij} \cdot (1 + \varepsilon_1) + \varepsilon_2 \quad (36)$$

where Y_{ij} is the observation of individual i at time t_j, $\hat{Y}_{ij}$ is the corresponding model prediction, and ε_1 and ε_2 are assumed to be independent and normally distributed random variables, respectively, with a zero mean and standard deviation (σ).
